# Development of a bead-based assay for detection of three banana-infecting viruses

**DOI:** 10.7717/peerj.13409

**Published:** 2022-05-26

**Authors:** Cheng-Ping Kuan, Chia-Hsin Tsai, Ching-Shan Tseng, Tso-Chi Yang

**Affiliations:** 1 Division of Biotechnology, Taiwan Agricultural Research Institute, Taichung, Taiwan; 2 Division of Plant Pathology, Taiwan Agricultural Research Institute, Taichung, Taiwan

**Keywords:** Banana bunchy top virus, Banana streak virus, Cucumber mosaic virus, Banana, Detection

## Abstract

**Background:**

Banana bunchy top virus (BBTV), cucumber mosaic virus (CMV) and banana streak virus (BSV) are important banana viruses, there are possible infections frequently with several viruses in field. Since the viruses are readily trasmitted in vegetative propagules, which pose a threat to banana production in banana-growing areas.

**Methods:**

A multiplex polymerase chain reaction (PCR) protocol combined with LiquiChip analysis to identify BSV, BBTV, and CMV, with consistent amplification of plant ubiquitin (*UBQ*), the banana plant messenger RNA used as a procedural control. Multiplex reverse transcription (RT)-PCR amplicons were extended by allele-specific primers, followed by hybridization with carboxylated microspheres containing unique fluorescent oligonucleotides, which were detected using the LiquiChip 200 workstation.

**Results:**

In this study, we aimed to develop a rapid, sensitive, and simultaneous detection method for BSV, BBTV, and CMV using a bead-based multiplex assay that can be applied in routine diagnosis. We demonstrated that this detection system was extremely efficient and highly specialized for differentiating individual in a mixture of viruses while being ten times more sensitive than traditional RT-PCR. The development of this method makes it feasible to detect banana viruses in field collected leaf samples.

## Introduction

Bananas are economically important fruit crops native to tropical and subtropical environments (FAOSTAT, 2018). Commercial banana plants are often propagated using suckers or tissue culture seedlings and are grown almost perennially. Multiple viruses known to naturally infect bananas have been characterized, including the banana streak virus (BSV), Banana bunchy top virus (BBTV), Banana bract mosaic virus, and Cucumber mosaic virus (CMV). These viruses are responsible for banana yield reduction and present obstacles to the international trade of banana plantlets (Kumar et al., 2015; Tripathi, Patil & Verma, 2016). Since they are readily transmitted in vegetative propagules (Kumar et al., 2015), they pose a threat to banana production in both virus-endemic areas and virus-free areas. Banana bunchy top disease is among the most severe banana viral diseases with an economic impact and is caused by a virus that belongs to the genus *Babuvirus* and family *Nanoviridae*. It is transmitted either by suckers or by the banana aphid, *Pentalonia nigronervosa*. Tissue-cultured plantlets could be important transmission sources due to latent infections or the lack of a good virus indexing scheme. Another major threat is BSV, a member of the genus *Badnavirus*, whose infection results in leaf streak disease, first recorded in 1986. BSV has been demonstrated to be transmitted by mealybugs but not by mechanical transmission (Kubiriba, Legg & Tushemereirwe Adipala, 2001). Prevalent infection by CMV, a member of the genus *Cucumovirus*, with mosaic symptoms, have been classified into three subgroups, namely IA, IB, and II by serology, which are also classified according to coat protein gene. CMV is reportedly transmitted by suckers and aphids. No banana species have so far been found to be resistant to any of these viruses; therefore, viral control strategies primarily focus on ensuring virus-free planting materials and crop protection using isolation for quarantine. The polymerase chain reaction (PCR) method is also used to identify viruses in banana (Mansoor et al., 2005). Meanwhile, multiplex PCR (Liu et al., 2012; Roy et al., 2005; Sharman, Thomas & Dietzgen, 2000) and loop-mediated isothermal amplification (LAMP) (Zhang et al., 2018) can detect several viruses in one tube and has been adopted for detecting some plant viruses. The development of BSV diagnostic methods has been significantly slower than those for other viruses that infect bananas, until the complete sequence of the BSV strain was reported (Geering et al., 2005). Another concern is that some BSV DNA has been integrated into the banana genome, and special identification measures are required, such as immunocapture PCR, rolling cycle amplification, which could distinguish between integrated BSV DNA in banana genome from episomal BSV (James et al., 2011; Le Provost et al., 2006).

This study describes the use of PCR for the detection of three characterized banana viruses simultaneously: one with an ssRNA genome (CMV) and two with ssDNA genomes (BBTV and BSV). LiquiChip assay, based on multi-analytic beads (Luminex xMAP technology), can be used for disease diagnosis and pathogen detection (Liu et al., 2011; Munro, Kuypers & Jerome, 2013; Oliver, Karem & Jacobson, 2003; van Brunschot et al., 2014; Xu et al., 2017); when combined with multiplex PCR, it can identify and quantify multiple viral pathogens (Dunbar, 2006; Taylor et al., 2001). In this study, we illustrate the application of a LiquiChip assay for the detection of BSV, BBTV, and CMV as well as an internal control present in a single tube. Sensitivity and specificity to the viruses are included in the assay evaluation, and then compared with a traditional RT-PCR assay.

## Materials and Methods

### Viruses and plants materials

Samples of Cavendish banana leaf tissues suspected to be infected with BSV, BBTV and CMV were collected in central and southern Taiwan. These leaf tissue samples included banana with symptoms and symptomless plants were tested for BSV, BBTV, and CMV by RT-PCR. All isolates used as reference viruses were kept in the greenhouse of the Taiwan Agricultural Research Institute (Kuan et al., 2019). Table 1 presents the primers used to amplify the specific regions of the BSV, BBTV and CMV genomes. Total nucleic acid was extracted from 100 mg infected banana leaves using Trizol Solution (Invitrogen, Waltham, MA, USA) following the guidelines provided by the manufacturer. The contaminated DNA was degraded by using the DNase I (Promega, Madison, WI, USA) treatment. Total RNA was extracted using Quick-RNA Mini-Prep kit (Zymo Research, Irvine, CA, USA) following the manufacturer’s instructions. RNA was quantified with a Nanodrop 2,000 (Thermo Fisher Scientific, Waltham, MA, USA) and these purified RNA was stored at −80 °C.

**Table 1:** Primers and MagPlex-TAG microsphere used in this study.

Target^a^	Target region^b^	Sequence (5’ to 3’) ^c^	Nucleotide position^d^	Amplicon Size (bp)	Bead address^e^
BBTV^a^	F	GGCAACAAGCCACGACTA	69–86	82	26
	R	CAGTAGGCTCAATCCTAAATACC	189–193		
	T	**TACATTCAACACTCTTAAATCAAA**TCGTCGTTAGGATCAATATTGGTTCC	88–113		
BSV^a^	F	GGAGCTACATTCCGAAAC	380–397	124	33
	R	GCTACAACTTCCTTGACC	486–503		
	T	**ACTACTTATTCTCAAACTCTAATA**GGAGCTACATTCCGAAAC	380–397		
CMV^a^	F	CCTCCTCGGATGCTAACTT	72–95	157	42
	R	GGTGGCTTTAGGGTAATAGATG	212–233		
	T	**CACTACACATTTATCATAACAAAT**CCTCCTCGGATGCTAACTT	72–95		
*Musa acuminate* UBQ^a^	F	TGCCTGCGATTCAGAACCT	1,061–1,079	316	36
	R	CACATTACCACCTAAGTCTCCTC	1,354–1,377		
	T	**ATTAAACAACTCTTAACTACACAA**TGC CTGCGATTCAGAACCT	1,061–1,079		

**Notes:**

a Banana Bunchy top virus (BBTV), Banana steak mottle virus (BSV), Cucumber mosaic virus (CMV), and the internal control (UBQ).

b Target genes were from Coat Protein gene. F, R, and T indicate forward primer, reverse primer, and TAG allele-specific primer extension primer, respectively.

c Underlined and bold indicates the MagPlex-TAG sequences provided by Luminex Corp.

d Position of primers for BBTV, BSV, CMV, and UBQ are based on accession numbers EU366171, FJ594891, AB261172, and HJQ853254, respectively.

e The MagPlex-TAG microsphere product number assigned by Luminex Corp.

### Primer design for PCR and TSPE/ASPE

The amplified sequences of BSV, BBTV, and CMV isolates were aligned to identify conserved regions (partial of coat protein gene) (Figs. S1, S2, and S3). In order to confirm whether the designed primers and probes were highly conserved regions, the nine identified isolates (variants) of BSV were included for the alignment (Fig. S2). PrimerPlex 2 (Primer Biosoft, Palo Alto, CA, USA) was used to design primers of target-specific primer extension/allele-specific primer extension (TSPE) primers in the multiplex PCR reactions (Table 1).

### Specificity of multiplex RT-PCR for BSV, BBTV and CMV

cDNA was prepared by reverse transcription (RT) of the total RNA as described in the user manual (Promega Co., Madison, WI, USA) in 20 μl reaction mixture which contained 1 μl of oligo-dT, 0.5 mM deoxynucleoside triphosphates (dNTPs), 4 μl 5X RT buffer, 0.01 M dithiothreitol, and 1 μg total RNA of banana leaves from healthy or virus-infected samples at 42 °C for 60 min for multiplex PCR. The RT-PCR analysis was conducted as described in our previous study (Kuan, Chang & Yang, 2018). Briefly, the PCR mix was conducted in a total 20 μl reaction consisted of 1 U Taq DNA polymerase (Platinum II; Invitrogen, Waltham, MA, USA), 2 μl of 10X PCR Buffer (Invitrogen, Waltham, MA, USA), 400 μM dNTPs, 0.4 μM each primer, 5–10 ng of template (RNA/cDNA) and ddH_2_O added to bring reaction to 20 μl. Amplification was conducted for 5 min at 94 °C, followed by 35 cycles at 94 °C for 30 s, 55 °C for 30 s, and 68 °C for 2 min each, and finally extended at 68 °C for 10 min. The RT-PCR products were identified using 2% agarose gel electrophoresis followed by ethidium bromide staining and observation under UV light. The PCR products of target fragment from BSV, BBTV and CMV were purified and cloned into pGEM-T Easy Vector (Promega, Madison, WI, USA) according to the kit manual. For optimization of RT-PCR, by varying the specific primer pair concentration ratios were 0.25–1.5 μM for BBTV and BSV, and 0.5–0.75 μM for CMV; for the UBQ internal control primers, they were 0.25–1.0 μM. Different combinations of the four primer set concentrations selected were then used to display clear virus-specific band densities.

### Target/Allele-specific primer extension (TSPE/ASPE)

The probe and primer pairs used in this study are shown in Table 1. The probe sequence has two parts of different functions as a primer in the TSPE reaction: the 3′ end (18 and 30 nts) of the probe serves as primer in the TSPE reaction and is complementary to certain specific gene sequences, while the 5′ end of it serves as a TAG sequence complementary to the anti-TAG sequence for xTAG magnetic bead hybridization. Allele-specific primer extension (ASPE) and hybridization to xTAG microspheres were carried out as protocol described by Kuan, Chang & Yang (2018) in 20 μl reaction volume, which contained 5.0 μl treated RT-PCR product, 1 × TSPE reaction buffer, 1.0 mM MgCl_2_, 0.05 μM for each TAG-ASPE primer, 0.75 U Tsp DNA polymerase, and 5 μM for each dNTP except for dCTP, and 5.0 μM biotin-14-dCTP (Invitrogen, Waltham, MA, USA). Thermocycling conditions of the TSPE reactions were 94 °C for 2 min, 30 cycles of 94 °C for 30 s, 55 °C for 30 s, and 74 °C for 1 min. The RT-PCR products were identified using 2% agarose gel electrophoresis followed by ethidium bromide staining and observation under UV light.

### LiquiChip analysis

The separate detection of TSPE products with biotin was based on the principle of flow cytometry, using specific hybridization with a set of magnetic beads. The xTAG magnetic bead sets were all provided by Luminex. Each set contains magnetic beads combined with complementary anti-TAG sequences tagged along with one of the TSPE probes attached to each additional specific TAG sequence. The allelic-specific of the hybridization reaction consists of a TSPE composition product and four Luminex xTAG mixed bead sets. Hybridization was performed in 96-well plates, and the volume of each reaction was 50 μL containing 25 μL xTAG bead mixture (2X Tm hybridization buffer: 0.4 M NaCl, 0.2 M Tris, 16% Triton X-100, pH 8.0) each μL contains 100 beads. Then adjust the total volume to 50 ul by adding of water to each well. The hybridization tests contained one TSPE extension product and mixed of each of the four MagPlex-TAG beads sets (Table 1). The hybridization conditions were conducted as described by Kuan, Chang & Yang (2018), which contained 25 μl xTAG beads mix (100 beads/μl) in 2 × TMAC buffer, 5 μl ASPE reaction, and 20 μl distilled water at 37 °C for 30 min. No-template control amplification was served as a background control. Use of magnetic separator (Luminex, Austin, Texas, USA) to separate the xTAG beads, and then wash the beads twice with 75 μl 1 × TMAC buffer. Resuspended xTAG beads in 75 μl 1 × TMAC buffer consist of 4 μg/mL streptavidin-R-phycoerythrin (Invitrogen), then incubate at 37 °C for 15 min. According to the system manual, analyze the final product on the LiquiChip 200 workstation at 37 °C, and the result was displayed as mean fluorescence intensity (MFI). For a given set of magnetic beads, any signal three times greater than the highest background MFI was regarded as a positive signal.

### Sensitivity test of LiquiChip assay

Equal amounts of BSV-, BBTV- and CMV-infected banana were mixed. To determine the detection limit of LiquiChip assay, nine ten-fold serial dilutions of mixed cDNA were prepared ranging from 10^−8^ to 10^−16^ g using DEPC-treated water, and tested in triplicate. For LiquiChip analysis, each reaction was the mean fluorescence intensity (MFI) reading of three replicate analysis samples. A positive reaction was defined when the mean fluorescence intensity (MFI) reading was three times above the background value, with mean values and error bars showed the standard deviations.

## Results

### Optimization of multiplex RT-PCR

**Figure 1:**
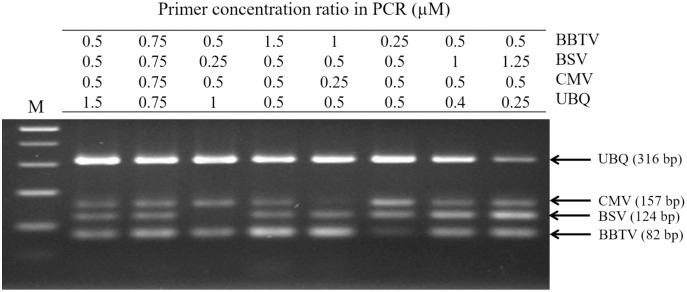
Polymerase chain reaction (PCR) detection for different concentration ratios of multiple primer sets. The assay was performed using the primer sets of banana bunchy top virus (BBTV), banana streak virus (BSV), cucumber mosaic virus (CMV) and the internal control (UBQ). The primer concentration ratios are list as the table. The PCR products of BBTV, BSV, CMV and Cyoxid are indicated by the arrows. Lane M: 100-bp DNA ladder.

**Figure 2:**
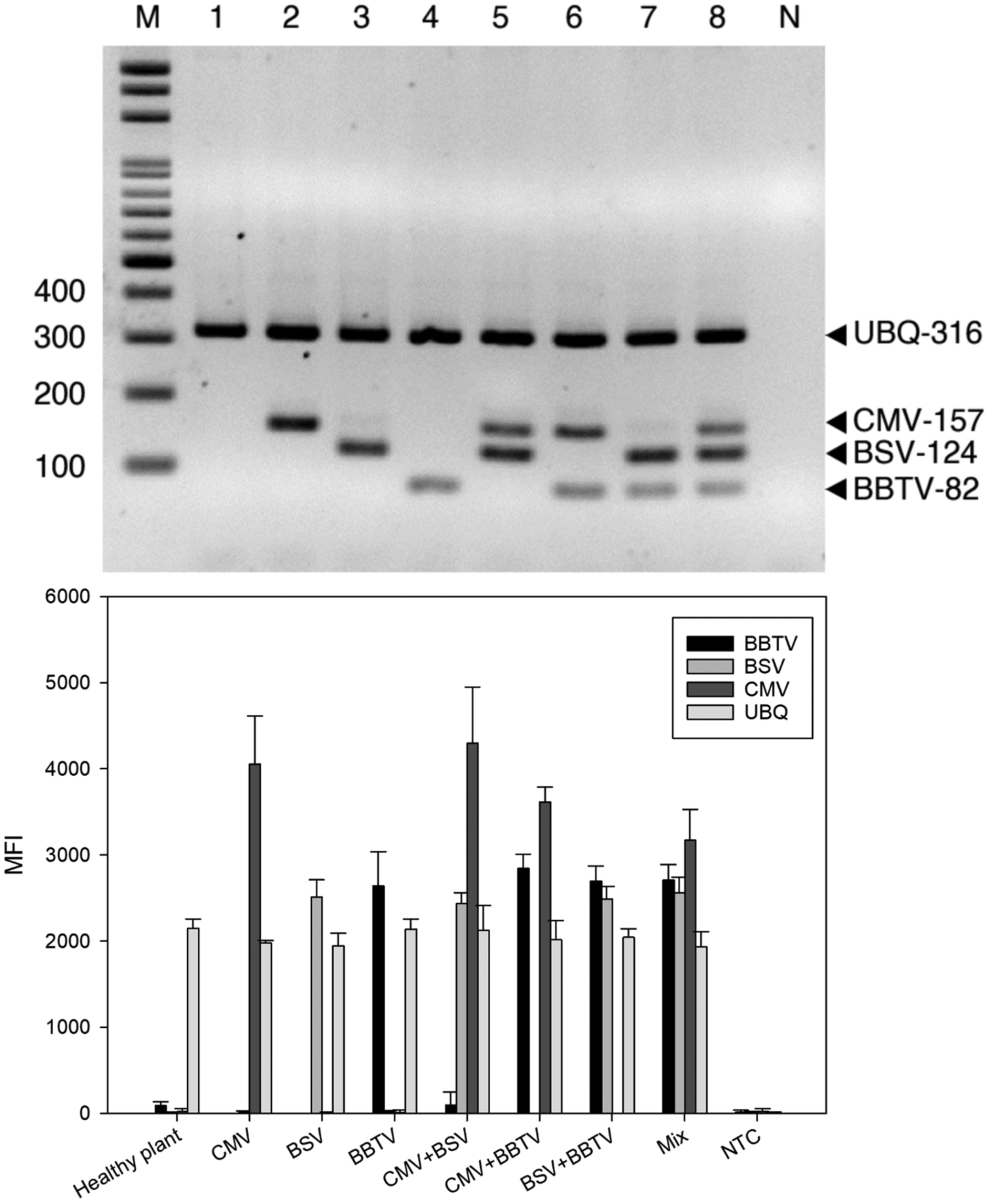
Specificity of reverse-transcription polymerase chain reaction (RT-PCR). RT-PCR (A) and LiquiChip assay (B) for the detection of banana bunchy top virus (BBTV), banana streak virus (BSV) and cucumber mosaic virus (CMV). Lanes 1, 2, 3, and 4 Healthy plant, CMV-, BSV-, and BBTV-infected total RNA, respectively; lane 5–7 CMV+BSV, BBTV+CMV, BSV+BBTV-infected RNA in equal amounts, respectively; lane 8 Mix (BBTV+ BSV + CMV)- infected RNA in equal amounts and lane M :100-bp DNA ladder. Each bar of LiquiChip represents the average MFI of three duplicate tests. NC represent no template in the reaction, respectively.

For RT-PCR, the optimal concentrations of each primer pair was set as 0.5 μM BBTV-specific primer, 1.25 μM BSV-specific primer, 0.5 μM CMV-specific primer, and 0.25 μM of plant ubiquitin (UBQ) as internal control primer. These pairs generated the expected bands of 316 bp for UBQ, 124 bp for BBTV, 157 bp for CMV, and 82 bp for BSV, respectively. Viral gene amplification was determined to be consistent when using the optimal primer ratio concentrations (Fig. 1). Evaluation of the RT-PCR products by gel electrophoresis showed that each primer pair is specific for its own target, since there was no cross-reaction with other target and non-target sequences (host plant nucleic acids) (Fig. 2A, lanes 2–4). The amplified products of BSV, BBTV, and CMV virus complementary DNA (cDNA) from all three viruses were the expected product sizes, although the PCR efficiency was slightly reduced (signified by the decrease in band intensity), by comparison to the LiquiChip assay as shown in Fig. 2A. As expected, the internal control was consistently amplified in all reactions with the exception of water or no-template control.

### Specificity of LiquiChip assay for BSV, BBTV, and CMV

To evaluate the specificity of the LiquiChip assay, template-specific primer extension (TSPE) reactions were performed utilizing all four TAG-TSPE primers on RT-PCR amplicons amplified from each virus, and with the internal controls. A LiquiChip 200 workstation was used to analyze the synthesized TSPE products hybridized with the beads. On utilizing a single template, elevated mean fluorescent intensity (MFI) values were obtained from each bead corresponding to its designated template (Fig. 2B). This result indicated that the TSPE primers exclusively amplified their targets. Analogous to multiplex RT-PCR, when a mixture of templates was used in the TSPE reaction, all three viruses were detected simultaneously with little loss in MFI values. Consistent co-amplification and detection of the internal controls suggest the reliability of the internal control primers and consistent presence of the internal control gene in the templates.

### Sensitivity of LiquiChip assay for the detection of banana viruses

**Figure 3:**
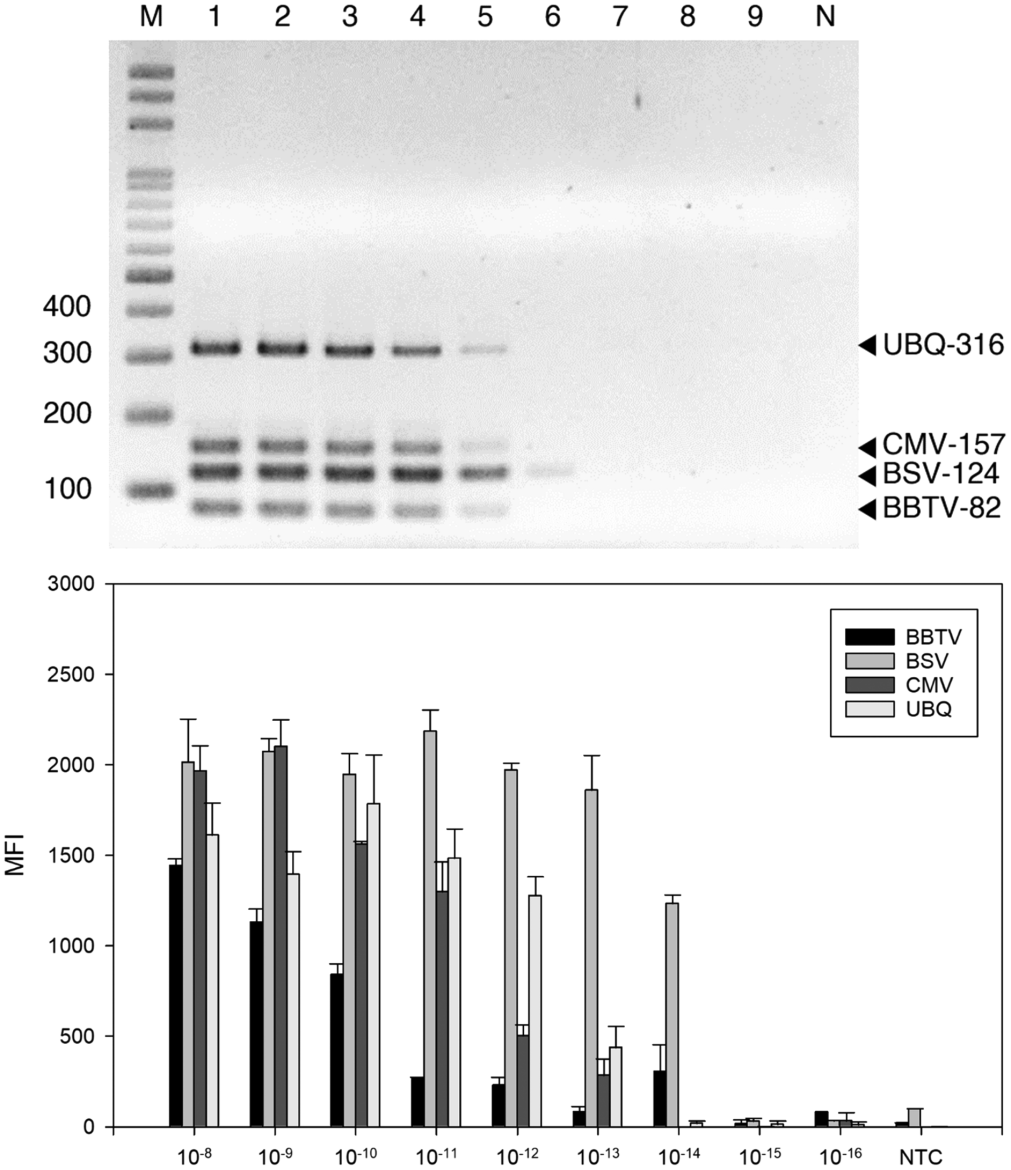
Sensitivity evaluation of reverse-transcription polymerase chain reaction (RT-PCR). RT-PCR (A) and LiquiChip assay (B) for detection of banana bunchy top virus (BBTV), banana steak virus (BSV) and cucumber mosaic virus (CMV). (A) Multiplex RT-PCR products amplified from a 10-fold serially diluted plasmid DNA. Lane M represents the 100-bp ladder; lanes 1 to 9 indicated 10^−8^ to 10^−16^ g serial dilutions, respectively. (B) Each bar represents the average MFI of the LiquiChip assay. N/NTC indicate no-template reaction, respectively. Error bars represent the standard deviations from three replicate reactions.

The detection threshold of the LiquiChip system was determined using ten-fold serial dilutions of BSV-, BBTV-, and CMV-infected banana RNA as template. The RT-PCR products were analyzed by gel electrophoresis; we found BBTV, CMV and BSV RNAs at 10^−12^ g and BSV RNAs at 10^−13^ g (Fig. 3A). The sensitivity of LiquiChip analysis was about 10 times higher than that of RT-PCR, and the detection limit of BSV, BBTV, and CMV was 10^−13^ g (Fig. 3B).

By blind-testing several banana plant samples with mosaic or striped leaves, the LiquiChip detection method was evaluated for its ability to simultaneously detect different viruses in banana samples from field. Forty banana plants collected from the field in mid and southern Taiwan were tested for the three viruses (Table 2). BBTV was the most common virus detected by LiquiChip in the 40 samples. Single infection of BBTV was detected in 20% of the samples, whereas multiple viruses were detected in 70% of the samples, which included BBTV+ CMV coinfection (Table 3). In our survey, no viruses were detected in 20% and 10% of the samples by RT-PCR and LiquiChip, respectively. The symptomatic samples that tested negative for any of the three viruses were probably infected with other viruses or their symptoms caused by mineral deficiencies.

**Table 2:** Screening results for 40 banana samples by RT-PCR and LiquiChip assay.

Isolate code number	Original hosts	Geographic Locations	Symptom^a^ (Mosaic-like)	RT-PCR^b^			LiquiChip^b^		
				BBTV	BSV	CMV	BBTV	BSV	CMV
S1	Cavendish	Taichung, Taiwan	+	−	−	+	−	−	+
S2	Cavendish	Taichung, Taiwan	+	+	−	−	+	−	+
S3	Cavendish	Taichung, Taiwan	+	+	−	−	+	−	+
S4	Cavendish	Taichung, Taiwan	+	−	−	+	+	−	+
S5	Cavendish	Taichung, Taiwan	+	+	−	+	+	−	+
S6	Cavendish	Taichung, Taiwan	+	+	−	−	+	−	+
S7	Cavendish	Taichung, Taiwan	+	+	−	−	+	−	+
S8	Cavendish	Taichung, Taiwan	+	+	−	−	+	−	+
S9	Cavendish	Taichung, Taiwan	+	+	−	−	+	−	+
S10	Cavendish	Taichung, Taiwan	+	+	−	−	+	−	+
S11	Cavendish	Taichung, Taiwan	+	+	−	−	+	−	+
S12	Cavendish	Taichung, Taiwan	−	−	−	−	−	−	+
S13	Cavendish	Nantou, Taiwan	−	−	−	−	+	−	+
S14	Cavendish	Nantou, Taiwan	−	−	−	−	+	−	+
S15	Cavendish	Nantou, Taiwan	−	−	−	−	+	−	+
S16	Cavendish	Nantou, Taiwan	−	−	−	−	+	−	−
S17	Cavendish	Nantou, Taiwan	+	+	−	−	+	−	+
S18	Cavendish	Nantou, Taiwan	−	−	−	+	+	−	−
S19	Cavendish	Nantou, Taiwan	−	−	−	−	+	−	+
S20	Cavendish	Nantou, Taiwan	−	−	−	−	+	−	−
S21	Cavendish	Kaohsiung, Taiwan	−	−	−	−	−	−	−
S22	Cavendish	Kaohsiung, Taiwan	+	+	−	−	+	−	+
S23	Cavendish	Kaohsiung, Taiwan	−	+	−	−	+	−	−
S24	Cavendish	Kaohsiung, Taiwan	−	−	−	−	−	−	−
S25	Cavendish	Kaohsiung, Taiwan	−	+	−	−	+	−	−
S26	Cavendish	Kaohsiung, Taiwan	+	+	−	−	+	−	+
S27	Cavendish	Kaohsiung, Taiwan	+	+	−	−	+	−	+
S28	Cavendish	Kaohsiung, Taiwan	+	+	−	−	+	−	+
S29	Cavendish	Kaohsiung, Taiwan	+	+	−	+	+	−	+
S30	Cavendish	Kaohsiung, Taiwan	+	+	−	−	−	−	+
S31	Cavendish	Kaohsiung, Taiwan	+	+	−	−	+	−	−
S32	Cavendish	Kaohsiung, Taiwan	+	+	−	−	+	−	−
S33	Cavendish	Kaohsiung, Taiwan	+	+	−	−	+	−	+
S34	Cavendish	Kaohsiung, Taiwan	+	+	−	−	+	−	+
S35	Cavendish	Kaohsiung, Taiwan	+	+	−	−	+	−	+
S36	Cavendish	Kaohsiung, Taiwan	+	+	−	−	+	−	+
S37	Cavendish	Kaohsiung, Taiwan	+	+	−	−	+	−	+
S38	Cavendish	Kaohsiung, Taiwan	+	+	−	−	+	−	+
S39	Cavendish	Kaohsiung, Taiwan	+	+	−	−	+	−	+
S40	Cavendish	Kaohsiung, Taiwan	−	+	−	−	+	−	−
BBTV-CK(+)				+	−	−	+	−	−
BSV-CK(+)				−	+	−	−	+	−
CMV-CK(+)				−	−	+	−	−	+
Healthy				−	−	−	−	−	−
CK(-)				−	−	−	−	−	−

**Notes:**

a +, positive (mosaic-like symptom); −, negative (without mosaic-like symptom).

b +, positive (visible band or MFI > 3*cutoff); −, negative (no visible band or MFI< cutoff).

**Table 3:** Number and percentage of banana samples tested positively by RT-PCR and LiquiChip.

	Number of sample detected (%)	
Virus	RT-PCR	LiquiChip
BBTV	26 (65%)	8 (20%)
CMV	3 (7.5%)	0
BBTV+ CMV	2 (5%)	28 (70%)
ND	9 (22.5%)	4 (10%)
Total	40 (100%)	40 (100%)

**Note:**

Banana bunchy top virus (BBTV), banana streak virus (BSV), and cucumber mosaic virus (CMV). ND: BBTV, BSV, and CMV were not detected from the GiantCavendish samples collected from fields of south and central Taiwan.

The results indicated that more than 90% of symptomatic banana plants were infected with either BBTV and CMV or a combination of these viruses. However, both RT-PCR and LiquiChip assays did not detect BSV in the field samples. (Tables 2 and 3).

## Discussion

Surveillance, monitoring, and diagnostics of target viruses based on visual assessment of symptoms is a rather difficult strategy to distinguish diseases in banana plants, particularly in early or mixed infections. BSV is one of the few plant viruses that have integrated sequences present in their host plant genome (Geering et al., 2005). In addition, the endogenous (integrated) BSV genome is usually a partial genome of the virus, containing incomplete functional genome. The noninfectious sequences probably are latent in plant genomes. However, endogenous BSV may get activated to become infectious under stress conditions or in cell culture (Dallot et al., 2001; Co^te et al., 2010; Gayral et al., 2008). It is difficult to identify infectious BSV only by PCR-based detection. RNA extraction may be contaminated with a small amount of genomic DNA; hence, there is a risk that integrated BSV DNA is also detected. DNase 1 treatment was therefore added in the field test to avoid the detection of residual genomic DNA during the extraction process. Meanwhile, for convenience, the leaf extracts containing RNA in our study have gone through a reverse transcription step. Zhang et al. (2018) also used RT-PCR to detect banana viruses. Moreover, the multiplex RT-PCR technique can be applied to detect the presence of BSV virus RNA transcript to prevent the occurrence of false positives. These findings show that the multiplex RT-PCR method can only detect transcripts from the virus; however, neither the endogenous BSV sequences nor healthy samples could be indexed. To prevent the detection of these aforementioned false positives, both BBTV and BSV were targeted as transcripts.

The LiquiChip detection technology, Luminex xMAP, requires a PCR amplification step, and its detection threshold can be confirmed by fluorescent signals. Primer and probe design plays a key role in successful amplification in RT-PCR (Roy et al., 2005). LiquiChip assay predominantly uses multiple virus probes in the detection of virus-infected plants through evaluation of MFI signals for each reaction. Furthermore, amplicon labeling only calls for one extra PCR step. The TSPE primers were able to amplify specific sequences in multiplex RT-PCR (Dunbar et al., 2003; Dunbar, 2006; Liu et al., 2011; Munro, Kuypers & Jerome, 2013; Oliver, Karem & Jacobson, 2003; van Brunschot et al., 2014; Xu et al., 2017). Mixed viral infections are frequent in perennial and vegetative plants. Likewise, the high prevalence of mixed infection of CMV with BBTV or BSV, which was also examined by serological tests and PCR-based methods (Fidan & Koc, 2019; Liu et al., 2012).

In banana plants, BBTV and CMV are mainly transmitted by vector insects, such as aphids. CMV infection is found in diverse hosts, not only in cultivated vegetables such as melon, pepper, tomato, and squash, but also in bananas through aphid transmission (Fidan & Koc, 2019). Further, BSV infection in bananas could occur through transmission by mealybugs in the field. In our studied, RT-PCR-based detection showed that BBTV (65%) and CMV (5%) were all from single infections. In contrast, LiquiChip detection demonstrated that only approximately 20% were single BBTV infections and most were multiple infections with BBTV and CMV (70%). A small number of samples were negative in RT-PCR but positive in LiquiChip assay and confirmed as BBTV or CMV by sequencing. This result indicated the limitation of RT-PCR detection. We found that in cases of multiple viral infections and uneven viral distribution in plants, it was not feasible to use RT-PCR to detect all viruses; however, LiquiChip had a higher detection rate (Wang et al., 2009). Furthermore, this study showed that BSV was not detected either using RT-PCR or LiquiChip. In addition, four suspicious BSV genomes (DNA) were detected in 40 banana samples by PCR method, and the positive PCR products were sequenced and confirmed to be BSV. However this BSV genome-containing virus may not be infectious, and the results may be due to endogenous viruses (Geering et al., 2005). The results showed that if the PCR method was used for field surveys, it may cause false positives for BSV. Banana RNA should still be tested to confirm that episomal BSV is actually present. The sensitivity of LiquiChip assay was 10–100-fold higher than that of RT-PCR assay. From the field survey, we found that the LiquiChip assay had superior detection rates compared to PCR. We believe that LiquiChip could be utilized for the early detection of viral infections when banana plants are still asymptomatic.

One of the major disadvantages of traditional nucleic acid-based assays is that each reaction can detect only one individual target pathogen. Although the LiquiChip assay could detect multiple banana viruses in a single tube, it still requires RNA purification during sample preparation, whereas ELISA is a simpler method as it only requires homogenized tissue crude saps. Although serological assays continue to be practical methods for plant virus detection and rapid sample indexing, obtaining specific and reliable monoclonal antibodies is often time-consuming. Nucleic acid-based assays do not require any antibody synthesis and vastly reduce the cost and time required for their development. From field survey results of banana viral infections, BBTV and CMV were the common viruses in field samples, which may be due to aphid transmission; BSV was less prevalent in symptomatic bananas.

Conventional multiplex RT-PCR is widely used to simultaneously detect multiple viruses; however, an agarose gel electrophoresis step is necessary after PCR. Furthermore, bands of similar sizes are difficult to resolve in gel electrophoresis. Real-time PCR could also detect multiplex viruses and have better sensitivity than LiquiChip (Munro, Kuypers & Jerome, 2013), but due to the limited number of fluorescence labels, simultaneous multiplex analysis could detect up to 4 or 5 targets, while Liquichip could increase to 100 targets in one tube reaction. Though LAMP has been used to detect multiple banana viruses (Zhang et al., 2018), it fails to detect and quantify a large number of samples.

## Conclusions

The LiquiChip system has the advantage of not only simultaneously identifying multiple banana viruses but also having sensitivity at least ten times higher than that of RT-PCR. More than 50 tests can be simultaneously performed for high throughput analysis and data acquisition using a 96 well plate LiquiChip system, and it is also possible to rapidly diagnose and quantify numerous nucleic acid targets. Moreover, we found that the LiquiChip assay applied in banana diseases could provide a quantitative diagnosis of mixed infections that could also be applied in the future in disease ecology and tracing (Dunbar, 2006). Moreover, a combination of LiquiChip and multiplex PCR methods allows concurrent identification of multiple infectious pathogens in a single reaction tube. Our field test results did not detect BSV infection in banana; this could be because there is no record of this virus in Taiwan to date. This study is the first report to describe a LiquiChip method that can simultaneously identify diverse banana viruses in a single reaction with a high degree of specificity and sensitivity. Additionally, we demonstrated the use of the LiquiChip assay to detect viral infection in banana leaf samples collected from field. Our developed novel LiquiChip technology could be utilized as a detection tool for field or quarantine testing.

## Supplemental Information

10.7717/peerj.13409/supp-1 Supplemental Information 1 Raw data for Figures 2 and 3.

10.7717/peerj.13409/supp-2 Supplemental Information 2 Primer and probe design for LiquiChip detection of Banana bunchy top virus. Accession numbers for the aligned isolates (top to buttom) are EU_366171, MG_545612.1, LC_481517.1, KX_779467.1, KT_923137.1, JQ_820467.1, JF_755978.1, HQ_616076.1, FJ_463044.1, AB_848053.1, AB_189068.1 were aligned and compared. Design of the primers (BBTV-F, BBTV-R) and probe (BBTV-P) were based on conserved sequences within the CP gene.

10.7717/peerj.13409/supp-3 Supplemental Information 3 Primer and probe design for LiquiChip detection of Banana streak virus. Accession numbers for the aligned isolates (top to buttom) are FJ594891, AY101189.1, FJ439815.1, FJ594892.1, HM447634.1, JQ346523.1, JQ911618.1, KF545102.1, KF548092.1, MH931389.1, AB_189068.1 were aligned and compared. Design of the primers (BSV-F, BSV-R) and probe (BSV-P) were based on conserved sequences within the RNase H gene.

10.7717/peerj.13409/supp-4 Supplemental Information 4 Primer and probe design for LiquiChip detection of Cucumber mosaic virus. Accession numbers for the aligned isolates (top to buttom) are AB261172.1, AB290155.1, AF5233431, DQ002881.1, DQ002882.1, JX865596.1, KF873615.1, KP713797.1, KT931619.1, LC368039.1, MG251400.1, MN593025.1, MW079239.1 were aligned and compared. Design of the primers (CMV-F, CMV-R) and probe (CMV-P) were based on conserved sequences within the CP gene.

## Additional Information and Declarations

### Data Availability

The following information was supplied regarding data availability: The raw measurements are available in the Supplemental Files.
