# Supplementary material for: Development of a bead-based assay for detection of three banana-infecting viruses
Source: PeerJ. 2022 May 26;10:e13409. doi: 10.7717/peerj.13409 (PMC9148560; doi:10.7717/peerj.13409)
Supplement: Supplemental Information 2 — Accession numbers for the aligned isolates (top to buttom) are EU_366171, MG_545612.1, LC_481517.1, KX_779467.1, KT_923137.1, JQ_820467.1, JF_755978.1, HQ_616076.1, FJ_463044.1, AB_848053.1, AB_189068.1 were aligned and compared. Design of the primers (BBTV-F, BBTV-R) and probe (BBTV-P) were based on conserved sequences within the CP gene. [file peerj-10-13409-s002.pdf]

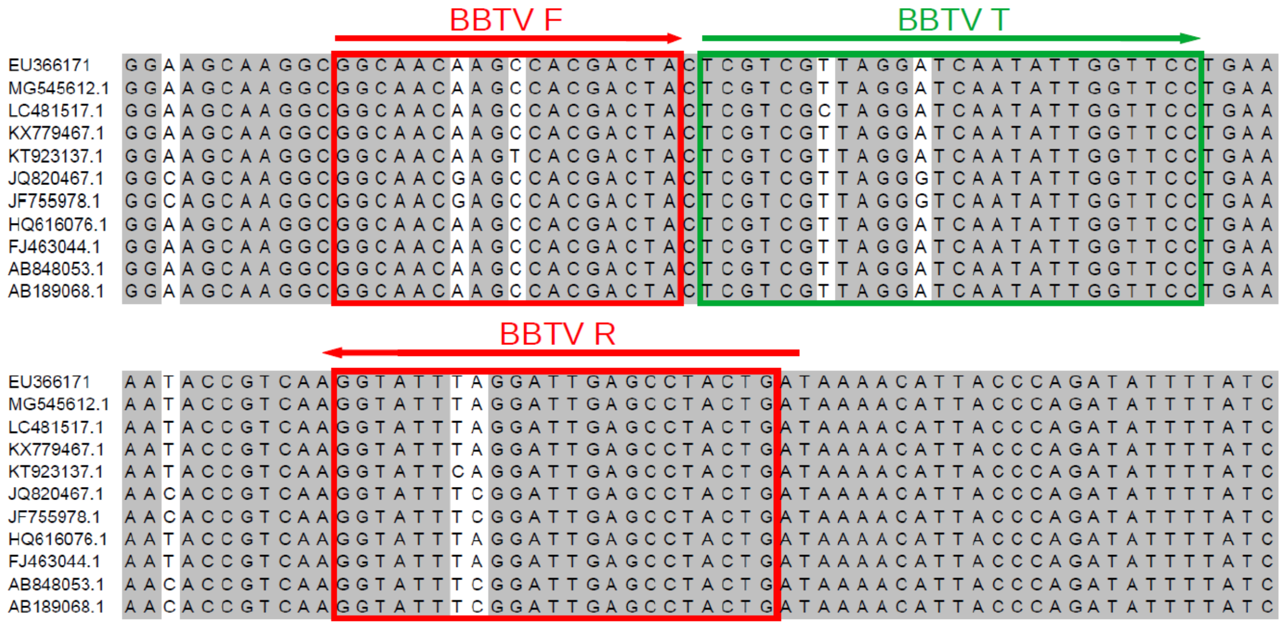

Fig. S1. Primer and probe design for LiquiChip detection of Banana bunchy top virus. Accession numbers for the aligned isolates (top to buttom) are EU_366171, MG_545612.1, LC_481517.1, KX_779467.1, KT_923137.1, JQ_820467.1, JF_755978.1, HQ_616076.1, FJ_463044.1, AB_848053.1, AB_189068.1 were aligned and compared. Design of the primers (BBTV-F, BBTV-R) and probe (BBTV-P) were based on conserved sequences within the CP gene.
