# Supplementary material for: Development of a bead-based assay for detection of three banana-infecting viruses
Source: PeerJ. 2022 May 26;10:e13409. doi: 10.7717/peerj.13409 (PMC9148560; doi:10.7717/peerj.13409)
Supplement: Supplemental Information 3 — Accession numbers for the aligned isolates (top to buttom) are FJ594891, AY101189.1, FJ439815.1, FJ594892.1, HM447634.1, JQ346523.1, JQ911618.1, KF545102.1, KF548092.1, MH931389.1, AB_189068.1 were aligned and compared. Design of the primers (BSV-F, BSV-R) and probe (BSV-P) were based on conserved sequences within the RNase H gene. [file peerj-10-13409-s003.pdf]

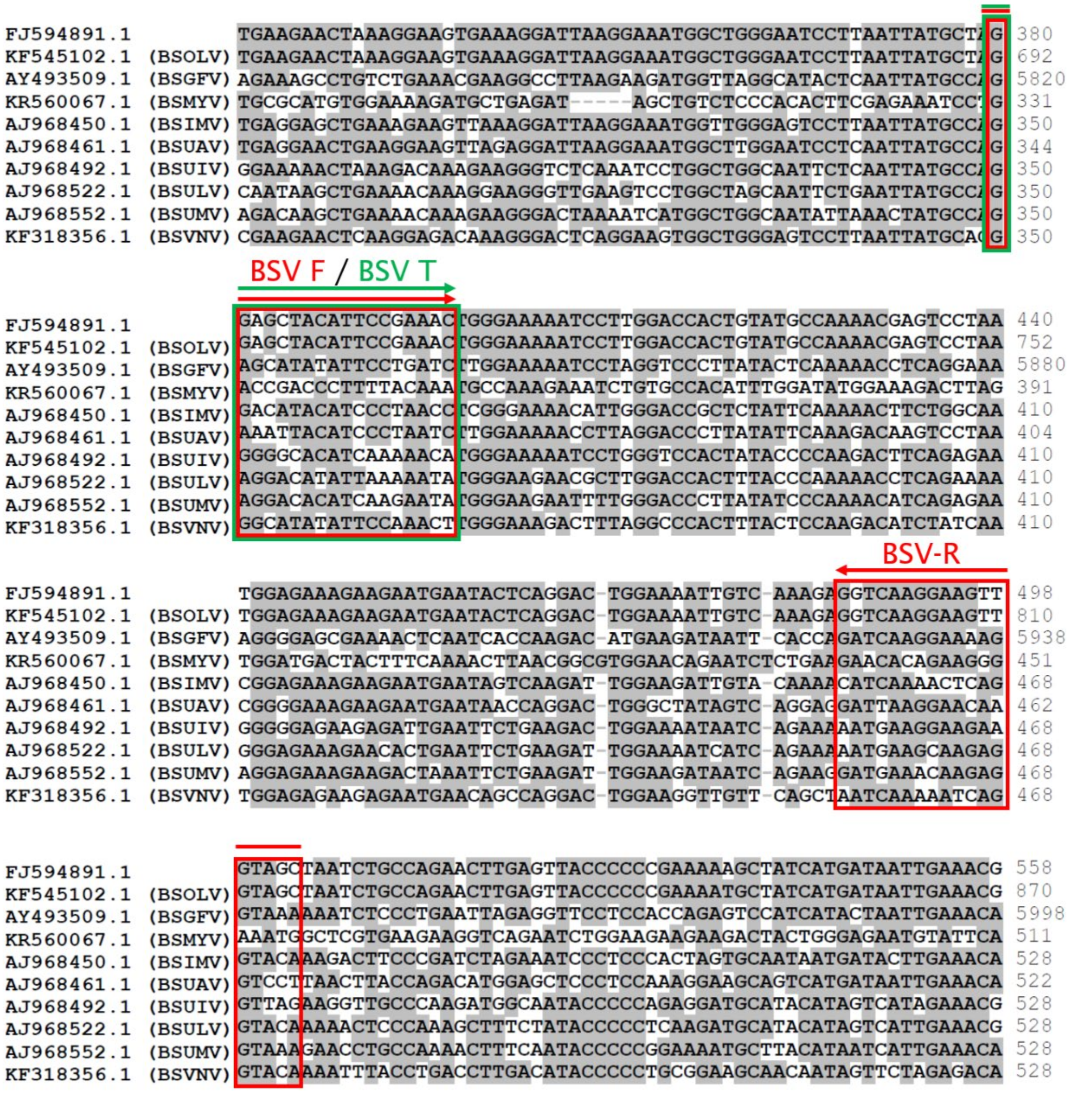

Figure S2 **Primer and probe design for LiquiChip detection of Banana streak virus.** Accession numbers for the aligned isolates (top to buttom) are FJ594891, AY101189.1, FJ439815.1, FJ594892.1, HM447634.1, JQ346523.1, JQ911618.1, KF545102.1, KF548092.1, MH931389.1, AB_189068.1 were aligned and compared. Design of the primers (BSV-F, BSV-R) and probe (BSV-P) were based on conserved sequences within the RNase H gene.
