# Supplementary material for: Development of a bead-based assay for detection of three banana-infecting viruses
Source: PeerJ. 2022 May 26;10:e13409. doi: 10.7717/peerj.13409 (PMC9148560; doi:10.7717/peerj.13409)
Supplement: Supplemental Information 4 — Accession numbers for the aligned isolates (top to buttom) are AB261172.1, AB290155.1, AF5233431, DQ002881.1, DQ002882.1, JX865596.1, KF873615.1, KP713797.1, KT931619.1, LC368039.1, MG251400.1, MN593025.1, MW079239.1 were aligned and compared. Design of the primers (CMV-F, CMV-R) and probe (CMV-P) were based on conserved sequences within the CP gene. [file peerj-10-13409-s004.pdf]

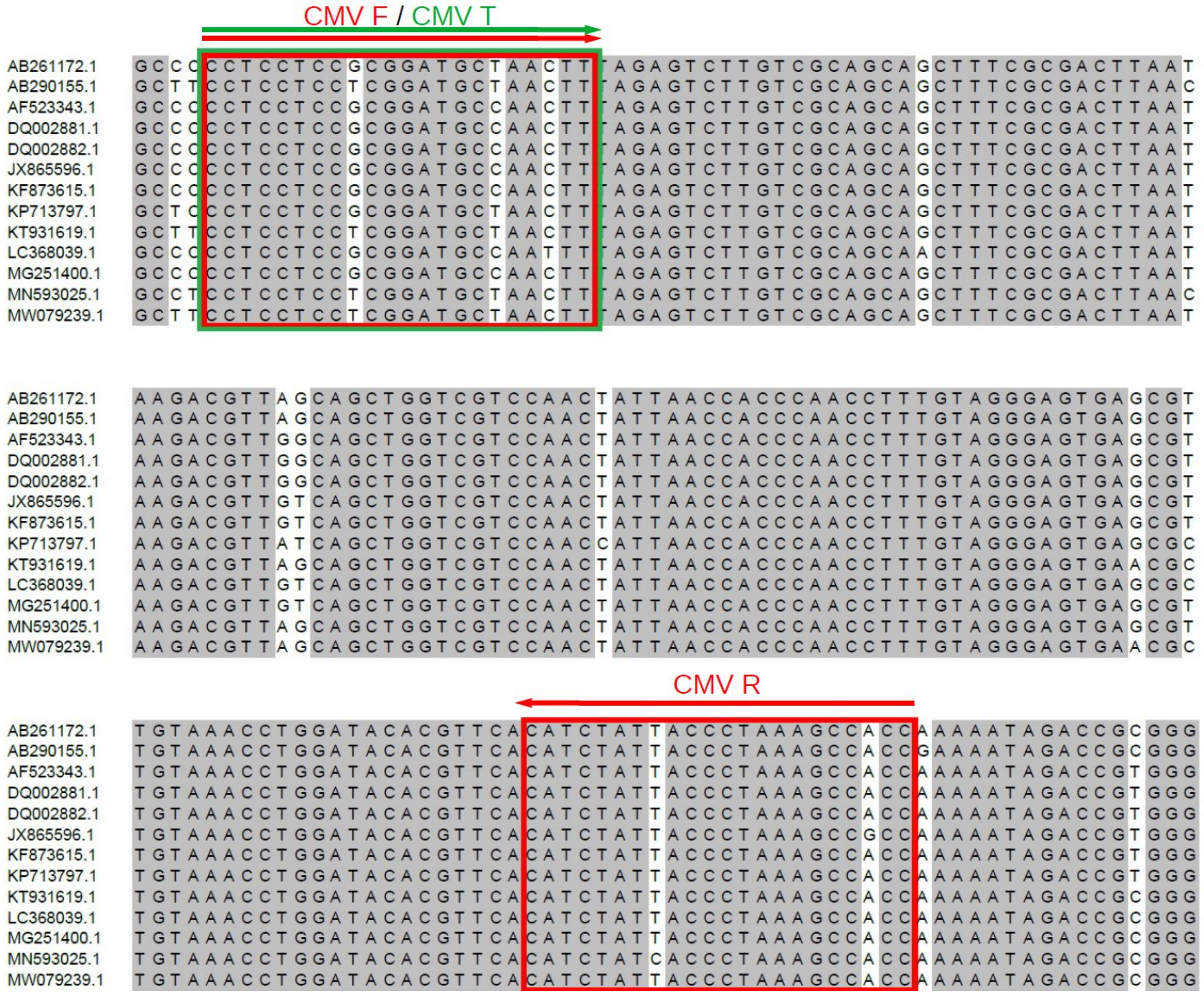

Figure S3 **Primer and probe design for LiquiChip detection of Cucumber mosaic virus**. Accession numbers for the aligned isolates (top to buttom) are AB261172.1, AB290155.1, AF5233431, DQ002881.1, DQ002882.1, JX865596.1, KF873615.1, KP713797.1, KT931619.1, LC368039.1, MG251400.1, MN593025.1, MW079239.1 were aligned and compared. Design of the primers (CMV-F, CMV-R) and probe (CMV-P) were based on conserved sequences within the CP gene.
